# HOXA1 is a radioresistance marker in multiple cancer types

**DOI:** 10.3389/fonc.2022.965427

**Published:** 2022-09-02

**Authors:** Lu He, Min Liang, Weisheng Guo, Jinquan Liu, Yi Yu

**Affiliations:** ^1^ Department of Radiotherapy, Affiliated Cancer Hospital and Institute of Guangzhou Medical University, Guangzhou, China; ^2^ Department of Oncology, The Fifth Affiliated Hospital of Guangzhou Medical University, Guangzhou Medical University, Guangzhou, China; ^3^ Key Laboratory of Biological Targeting Diagnosis, Therapy and Rehabilitation of Guangdong Higher Education Institutes, The Fifth Affiliated Hospital of Guangzhou Medical University, Guangzhou, China

**Keywords:** radioresistance, HOXA1, biomarker, transcription factor, radiotherapy

## Abstract

Radiotherapy is an important therapeutic method for patients with cancer. However, radioresistance can cause treatment failure. Thus, there is an urgent need to investigate mechanisms of radioresistance and identity markers that could be used to predict radioresistance and prognosis of post-radiotherapy cancer patients. In the present study, we propose HOXA1 as a candidate biomarker of intrinsic radioresistance in multiple cancer types. By analyzing data from The Cancer Genome Atlas (TCGA), we found that HOXA1 was aberrantly upregulated in multiple cancers, and that elevated HOXA1 was significantly associated with poor prognosis of post-radiotherapy head and neck squamous cell carcinoma (HNSCC) and low-grade glioma (LGG) patients. Correlation analysis showed that HOXA1 expression was positively correlated with expression of EGFR, CDK6, and CAV1, which have been reported to enhance radioresistance. In addition, gene set enrichment analysis (GSEA) showed that the oxidative phosphorylation gene set was negatively enriched in HOXA1 high-expression samples in both HNSCC and LGG. Moreover, immunohistochemical assays indicated that high HOXA1 expression was significantly correlated with a high recurrence rate of nasopharyngeal carcinoma (NPC) after radiotherapy. Further *in vitro* experiments demonstrated that HOXA1 knockdown markedly attenuated the DNA repair capacity of NPC cells and sensibilized NPC cells to irradiation. Taken together, the results of this study demonstrate that HOXA1 has potential to be a predictive marker for radioresistance and post-radiotherapy prognosis that could help to guide individualized treatment in multiple cancer types.

## Introduction

About half of cancer patients receive radiotherapy at some point after their diagnosis of cancer (1). For patients with nasopharyngeal carcinoma (NPC), radiotherapy is the preferred radical treatment method because of the anatomical location of the cancer (2). In addition, radiotherapy is an important adjuvant therapy method used to prevent in situ recurrence of many types of cancer, including head and neck squamous cell carcinoma (HNSCC) and glioma (3, 4).

Recent advances in radiotherapy have led to significant benefits for many cancer patients, enabling improved tumor control with reduced toxic effects (5). However, the response to radiotherapy varies owing to inter-individual differences in the radiosensitivity of tumor cells. For example, residual tumor cells might persist in some patients even after irradiation with a total dose of 80 Gy, whereas other patients might achieve complete remission after only 40 Gy irradiation (6). In addition, radioresistance, which induces local treatment failure and results in residual or recurrent tumors, remains an important problem in radiotherapy. For NPC, 7.4–14% of patients who receive radiotherapy develop local recurrence or persistent disease (7, 8). Therefore, it is important to understand the mechanisms of tumor radioresistance to enable development of resistance-reversal strategies and identification of radioresistance biomarkers that could be used to guide individualized radiotherapy.

The homeobox genes encode a highly conserved subgroup of homeobox transcription factors, which regulate multiple processes including development, differentiation, apoptosis, motility, angiogenesis, and even carcinogenesis (9). Homeobox A1 (HOXA1) is a member of the A cluster of homeobox transcription factors. HOXA1 expression is extremely low during normal growth and differentiation but is detectable in a variety of human cancer lesions (10). Studies have reported that HOXA1 functions as an oncogene. In breast cancer, HOXA1 mediates oncogenic transformation via activation of the p44/42 MAP kinase pathway and is related to endocrine therapy resistance (11, 12). Moreover, elevated HOXA1 expression promotes cell proliferation in gastric cancer and drives tumor growth and metastasis in melanoma (13, 14). In addition, aberrant upregulation of HOXA1 is correlated with poor prognosis of patients with hepatocellular carcinoma, prostate cancer, and HNSCC (15–18). Although these studies suggest that upregulation of HOXA1 promotes cancer progression, the role of HOXA1 in determining the biological properties of malignant tumors is not fully understood, nor has the correlation between HOXA1 expression and cancer radioresistance been reported.

In this study, we identified HOXA1 as a new intrinsic radioresistance marker of multiple cancer types. Reanalysis of data from The Cancer Genome Atlas (TCGA) and the results of *in vitro* experiments suggest that high expression of HOXA1 contributes to radioresistance in NPC, HNSCC, and LGG.

## Materials and methods

### Cell culture and RNA interference

CNE1 (CVCL_6888) and HNE1 (CVCL_0308) human NPC cell lines were obtained from the State Key Laboratory of Oncology in South China, Sun Yat-sen University Cancer Centre. The cells were confirmed to be negative for mycoplasma with a PCR Mycoplasma Detection Kit (Beyotime, Shanghai, China) and cultured in RPMI 1640 supplemented with 10% fetal bovine serum (Thermo Fisher Scientific, Waltham, MA, USA) at 37°C under 5% CO2.

For RNA interference, cells were transfected with a chemically synthesized scrambled short interfering RNA (siRNA) or HOXA1 siRNA using Lipofectamine 3000 (Thermo Fisher Scientific) in accordance with the manufacturer’s instructions. The HOXA1 siRNA was synthesized by Ribobio Co. Ltd., and the sequence (sense: 5′ GUUCCUUUCAGAUGACCUU 3′) was as previously reported. The scrambled control siRNA was purchased from Ribobio Co. Ltd. The knockdown efficiency was validated by quantitative PCR (qPCR) assays and confirmed to be more than 75%.

### Cell irradiation and clonogenic survival assay

Cell irradiation and clonogenic survival assays were performed as previous reported (19). The indicated cells were seeded into 3.5cm culture dishes at different densities (100, 100, 200, 1000, 10000 and 100000 cells for 0, 0.5, 1, 2, 4, 6 and 8 Gy irradiation, respectively). After the cells became adherent, the cells were irradiated at defined doses using a Rad Source (Rad Source Tech, Suwanee, GA, USA) R2000 X-ray irradiator (1.1 Gy/min., 160 kV, 25 mA, 0.3 mm copper filters). After 14 days incubation, the cells were fixed and stained with crystal violet stain. Then the Colonies were scored. The plating efficiency (PE) was calculated by dividing the number of counted colonies by the number of cells plated. The surviving fractions (SF) were then calculated by dividing the PE by the PE of the non-irradiated control. The radiation dose-clonogenic survival curves were fit to a linear-quadratic model as previously described. The curves were compared using the extra sum-of-squares F test in GraphPad Prism 8.0 (GraphPad, La Jolla, CA, USA).

### Patient specimens

Paraffin-embedded NPC specimens used for immunohistochemistry assays were obtained from 70 NPC patients without distant metastases who had undergone conventional radiotherapy at the Affiliated Cancer Hospital of Guangzhou Medical University from 2013 to 2015 (**Supplementary Table 1**). All patients were monitored for more than 5 years, during which time 20 of them experienced local recurrence. This study was approved by the Ethics Committee of Affiliated Cancer Hospital of Guangzhou Medical University (ZN2022-22).

### Immunohistochemistry assays

Formalin-fixed, paraffin-embedded NPC tissue specimens were analyzed by immunohistochemistry as previously described (20). Briefly, a primary antibody against HOXA1 (ab230513; rabbit polyclonal; working dilution 1:100; Abcam, Cambridge, UK) was used for immunohistochemistry assays, and a non-biotin horseradish peroxidase detection system (DAKO, Glostrup, Denmark) was used to detect the expression level of HOXA1 protein. Both the extent and intensity of immunostaining were taken into consideration when analyzing the data. The intensity of staining was scored from 0 to 3, and the extent of staining was scored from 0% to 100%. The final quantitation of each stain was obtained by multiplying the two scores. HOXA1 expression was classified as high if the score was higher than 1.5, whereas scores of 1.5 or less indicated low expression.

### Cell proliferation assays

Three thousand of the indicated cells were seeded per well in 96-well plates. after 24h, 48 and 72h of incubation, cell proliferation was assessed using CCK8 reagents (Dojindo, Kumamoto, Japan) according to the manufacturer’s instructions. Three replicates were used for each experiment.

### Immunofluorescence assays

Cells were trypsinized and seeded on glass chamber slides. After 24 h, the cells were irradiated at a dose of 2 Gy. Immunofluorescence analysis of γH2AX was performed 0.5 and 24 h after irradiation. The immunofluorescence analysis was performed as previously described (19). Briefly, the cells were fixed with cold methanol for 10 min, followed by blocking in blocking buffer for 30 min. Next, the cells were incubated with a rabbit monoclonal antibody against phospho-H2A.X (1:200; Cell Signaling Technology; 20E3, #9718) and anti-rabbit Alexa 555-conjugated secondary antibody (Thermo Fisher Scientific). Then, nuclei were counterstained with DAPI solution (Thermo Fisher Scientific), and the slides were mounted with antifade mounting solution (Thermo Fisher Scientific). Images were taken using a Zeiss LSM800 confocal imaging system. Cells with more than ten γH2AX foci were defined as γH2AX-positive cells. Five random fields were examined to estimate the number of γH2AX-positive cells per field for each slide.

### Real-time qPCR

Total mRNA of cells was isolated using TRIzol reagent (Thermo Fisher Scientific). One microgram of RNA was used for cDNA synthesis with a Transcriptor First Stand cDNA Synthesis Kit (Thermo Fisher Scientific). Real-time qPCR assays were performed according to the manufacturer’s instructions using SYBR Green mix and LightCycler480 II system (Roche, Basel, Switzerland). The results were normalized to GAPDH expression using the 2-ΔΔCT method. Samples were run in triplicate. The following primers were used: HOXA1 forward, TCCTGGAATACCCCATACTTAGC; HOXA1 reverse, GCACGACTGGAAAGTTGTAATCC. GAPDH forward, GGAGCGAGATCCCTCCAAAAT; GAPDH reverse, GGCTGTTGTCATACTTCTCATGG. EGFR forward, AGGCACGAGTAACAAGCTCAC; EGFR reverse, ATGAGGACATAACCAGCCACC. CDK6 forward, CCAGATGGCTCTAACCTCAGT; CDK6 reverse, AACTTCCACGAAAAAGAGGCTT. CAV1 forward, GCGACCCTAAACACCTCAAC; CAV1 reverse, ATGCCGTCAAAACTGTGTGTC.

### Public data analysis

TCGA and Genotype-Tissue Expression data were acquired from UCSC Xena (21). Expression and survival data for HNSCC and low-grade glioma (LGG) cases were obtained from the TCGA Research Network (http://cancergenome.nih.gov/). In the HNSCC cohort, 295 patients had undergone postoperative radiotherapy, 149 patients did not undergo radiotherapy, and relevant information was lacking for 56 patients. In the LGG cohort, 302 patients had undergone postoperative radiotherapy, 178 patients did not undergo radiotherapy, and relevant information was lacking for 30 patients. We only included patients for whom adequate information was available in this study. Expression data were analyzed using R (version 3.6.3, http://www.r-project.org/) and R package DESeq2 (22); and gene ontology (GO) analysis and gene set enrichment analysis (GSEA) were performed using R and R package ClusterProfiler (23). The protein–protein interaction (PPI) network of the overlapping different expression genes was constructed based on the STRING online database (24).

### Statistics

At least three replicates of each experiment were conducted. Student’s t-tests or chi-squared tests were used to compare differences, as appropriate. Survival curves were constructed using the Kaplan–Meier method and analyzed by log-rank test or Cox regression model. The correlation coefficients between HOXA1 and EGFR, HOXA1 and CDK6, and HOXA1 and CAV1 expression levels were calculated using Pearson’s correlation test. The two-tailed chi-squared test was used to analyze the association of HOXA1 expression with clinical parameters. Dose–survival curves were compared using the extra sum-of-squares F-test in GraphPad Prism 8.0 (GraphPad Software). P<0.05 was considered to indicate a significant difference.

## Results

### Pattern and prognostic significance of HOXA1 expression in human cancers

**Figure 1:**
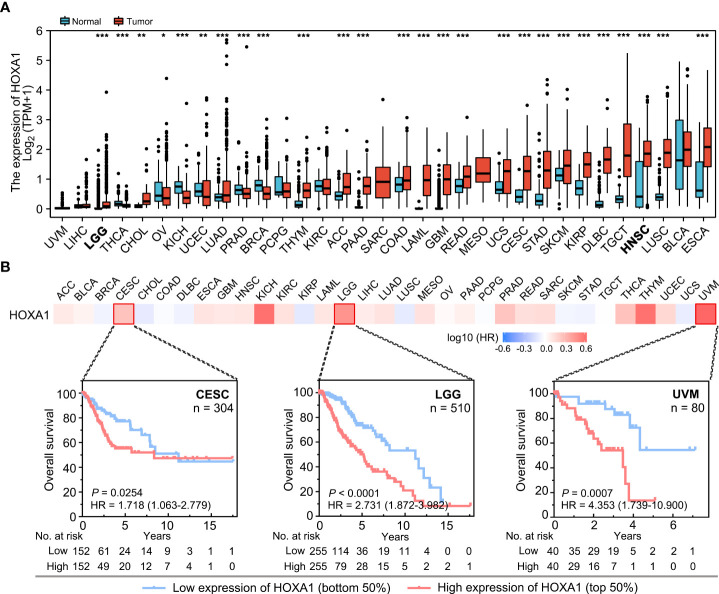
HOXA1 expression and the prognostic significance of HOXA1 in different cancer types. **(A)** HOXA1 expression in different cancer types. Data are from TCGA. **(B)** Heatmap of hazard ratios for overall survival according to HOXA1 expression in different cancers. Kaplan–Meier survival curves for CESC, LGG, and UVM are shown. *P < 0.05; **P < 0.01; ***P < 0.001.

Expression analysis based on TCGA data indicated that HOXA1 was significantly upregulated in most cancer types (**Figure 1A**), including CESC (cervical squamous cell carcinoma and endocervical adenocarcinoma), LGG (low-grade glioma), GBM (Glioblastoma multiforme), and HNSCC. The results also indicated that HOXA1 was downregulated in BRCA (breast-invasive carcinoma), KICH (kidney chromophobe), OV (ovarian serous cystadenocarcinoma), PRAD (prostate adenocarcinoma), THCA (thyroid carcinoma), and UCEC (uterine corpus endometrial carcinoma) compared with adjacent non-tumor tissues (**Figure 1A**).

Moreover, we analyzed the prognostic value of HOXA1 expression in different human cancers using TCGA data (**Figure 1B**). We found that high HOXA1 expression was a risk factor for poor prognosis in multiple cancers, including CESC, LGG, and UVM (uveal melanoma).

Taken together, these results suggest that high HOXA1 expression could serve as a biomarker for poor prognosis in several cancers, and that the prognostic significance of HOXA1 expression depended on the cancer type.

### High HOXA1 expression predicted radioresistance in HNSCC and LGG

**Figure 2:**
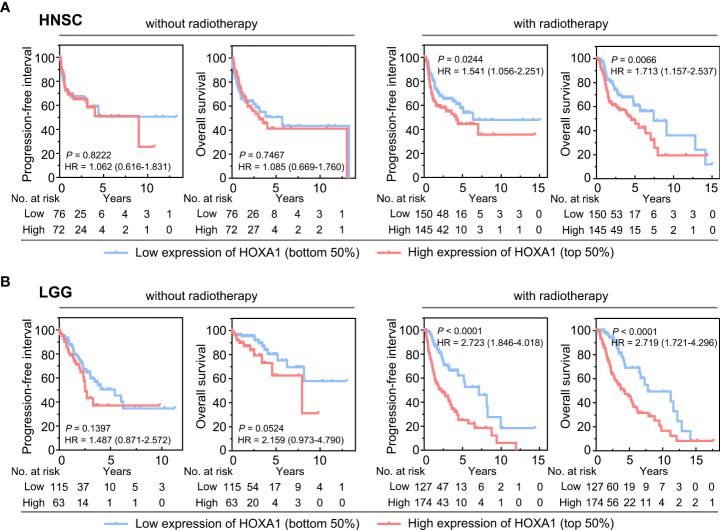
HOXA1 expression had prognostic significance in HNSCC and LGG patients receiving postoperative radiotherapy. **(A)** Overall survival (OS) and progression-free interval (PFI) curves of HNSCC patients with and without postoperative radiotherapy. **(B)** OS and PFI curves of LGG patients with and without postoperative radiotherapy.

We also found that high HOXA1 expression was associated with radioresistance in HNSCC and LGG. Based on TCGA data, we found that high HOXA1 expression was correlated with poor prognosis and a high recurrence rate in HNSCC and LGG patients who had undergone postoperative radiotherapy (**Figures 2A, B**). However, HOXA1 expression did not have prognostic significance in HNSCC and LGG patients that did not undergo radiotherapy (**Figures 2A, B**). These results demonstrate that HOXA1 promotes radioresistance and increases local recurrence rates in patients with certain tumor types.

### Transcriptomes of HNSCC and LGG tissues with varying HOXA1 expression

**Figure 3:**
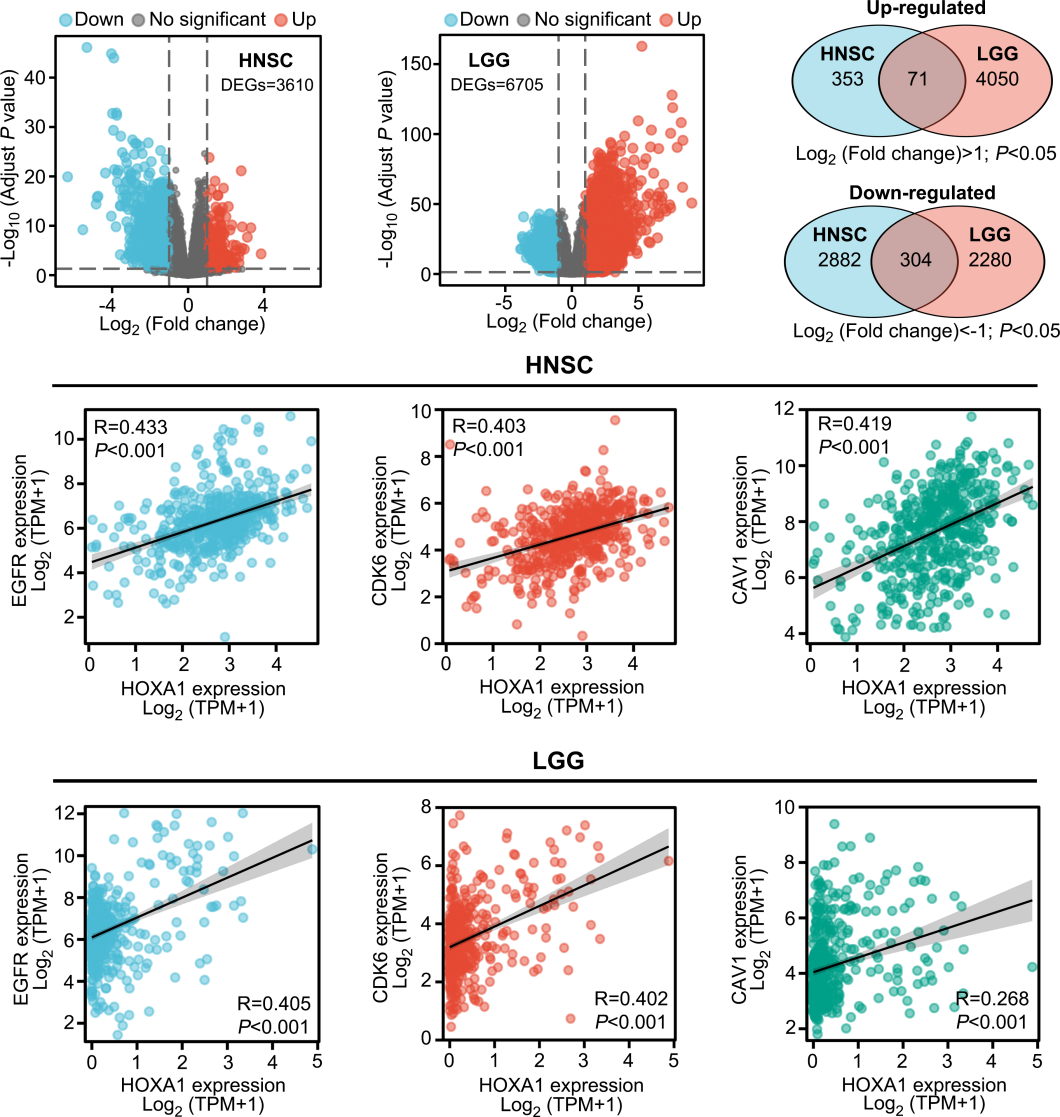
Transcriptomes of HNSCC and LGG tissues with varying HOXA1 expression. **(A, B)** Volcano plot showing the DEGs (*P* < 0.05, log_2_ fold change<−1 or log_2_ fold change >1) between high HOXA1 expression (top 25%) and low HOXA1 expression (bottom 25%) tumor tissue samples from HNSCC or LGG patients. **(C)** Venn plot depicting the overlapping genes with similar trends of expression changes in HNSCC and LGG samples with varying HOXA1 expression. **(D)** HOXA1 expression was significantly correlated with EGFR, CDK6, and CAV1 expression in HNSCC tissues. **(E)** HOXA1 expression was significantly correlated with EGFR, CDK6, and CAV1 expression in LGG tissues.

HOXA1 has been identified as a transcription factor that regulates the expression of various genes (25). To further investigate the downstream genes of HOXA1 and the molecular mechanism by which HOXA1 affects radiosensitivity, we identified the differentially expressed genes (DEGs) between samples with high (highest quartile) and low (lowest quartile) HOXA1 expression in HNSCC and LGG based on TCGA data, respectively. We found 424 significantly upregulated (log2 fold change>1; P<0.05) and 3186 significantly downregulated (log2 fold change<−1; P<0.05) genes in the HNSCC cohort (**Figure 3A**). On the other hand, we found 4121 significantly upregulated (log2 fold change>1; P<0.05) and 2584 significantly downregulated (log2 fold change<−1; P<0.05) genes in the LGG cohort (**Figure 3B**). Among these DEGs, we identified 71 overlapping upregulated DEGs and 304 downregulated DEGs (**Figure 3C**). Based on these data, we found that HOXA1 expression was significantly correlated with some well-reported radioresistance-associated genes including EGFR, CDK6, and CAV1 (**Figures 3D, E**) (26–28). This suggests that the overlapping DEGs could be key downstream genes of HOXA1 that affect the radiosensitivity of cancer cells.

### HOXA1-associated signaling pathway

**Figure 4:**
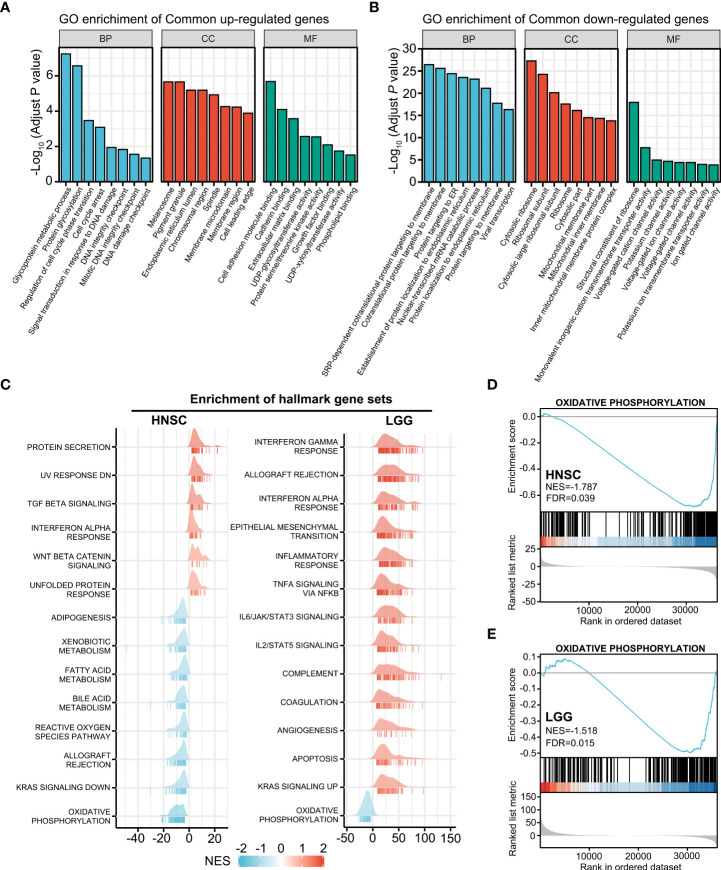
HOXA1-associated signaling pathway. **(A)** GO enrichment analysis of overlapping upregulated genes (*P*< 0.05) of the HNSCC and LGG cohorts. **(B)** GO enrichment analysis of the overlapping downregulated genes (*P* < 0.05) of the HNSCC and LGG cohorts. **(C)** Ridgeline plots depicting the significantly enriched signaling pathways involving the HOXA1-associated DEGs in HNSCC and LGG as revealed by GSEA performed using 50 hallmark gene sets. **(D, E)** GSEA plots showing that the oxidative phosphorylation gene set was negatively enriched in HNSCC and LGG samples with high HOXA1 expression.

To investigate the HOXA1-associated signaling pathway, we performed GO enrichment analysis on the overlapping DEGs (P<0.05) of the HNSCC and LGG cohorts. Several terms associated with DNA damage and repair, including signal transduction in response to DNA damage, DNA integrity checkpoint, and DNA damage checkpoint, were enriched in the overlapping upregulated genes (**Figure 4A**), whereas terms including co-translational protein targeting to membrane, protein targeting to ER, and protein localization to endoplasmic reticulum were enriched in the overlapping downregulated genes (**Figure 4B**).

We also performed GSEA using MSigDB hallmark gene sets (**Figure 4C**). The oxidative phosphorylation gene set was negatively enriched in HOXA1 high-expression samples in both HNSCC and LGG cohorts (**Figure 4D, E**). Previous studies have reported that enhanced oxidative phosphorylation could enhance the radiosensitivity of glioma cells (29). Hence, HOXA1 could induce radioresistance of cancer cells via inhibition of oxidative phosphorylation. In addition, we constructed a PPI network containing the most significant overlapping DEGs (log2 fold change>1 or log2 fold change<−1; P<0.05) to identify the most significant clusters of the overlapping DEGs of HNSCC and LGG (**Supplementary Figure 1**).

### Knockdown of HOXA1 enhanced the radiosensitivity of NPC cells

**Figure 5:**
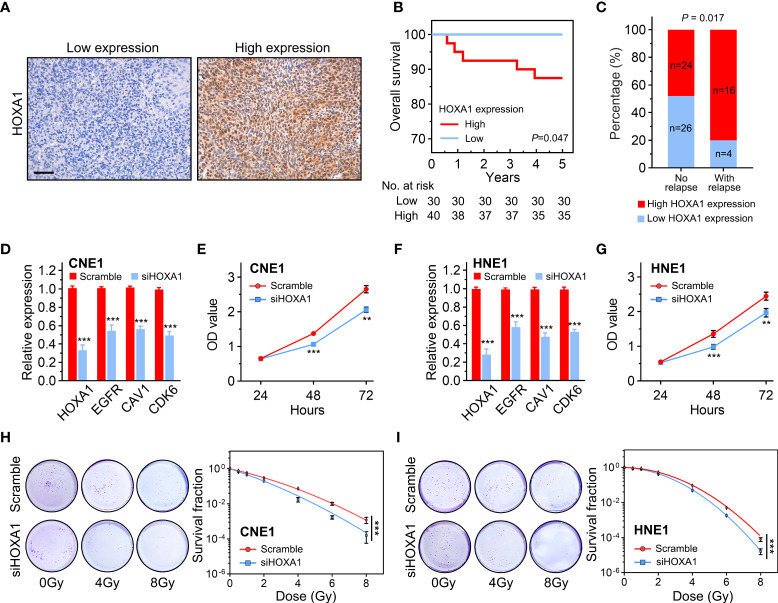
HOXA1 enhanced the radioresistance of NPC cells. **(A)** Representative immunohistochemistry images showing low and high HOXA1 expression in NPC tissues. Scale bars, 50 μm. **(B)** Overall survival curves of NPC patients. **(C)** Cumulative bar chart showing that high HOXA1 expression was correlated with high risk of local relapse in NPC patients. **(D)** knockdown of HOXA1 expression downregulated EGFR, CAV1 and CDK6 expression in CNE1 cells. **(E)** knockdown of HOXA1 expression reduced the proliferation of CNE1 cells. **(F)** knockdown of HOXA1 expression downregulated EGFR, CAV1 and CDK6 expression in HNE1 cells. **(G)** knockdown of HOXA1 expression reduced the proliferation of HNE1 cells. **(H, I)** Dose-survival curves showing that knockdown of HOXA1 expression enhanced the radiosensitivity of CNE1 and HNE1 NPC cells. **P < 0.01; ***P < 0.001.

Radiotherapy is the principal treatment modality for NPC. To determine whether HOXA1 affected the radiosensitivity of NPC, we performed immunohistochemistry assays to examine HOXA1 expression in 20 NPC samples from patients who experienced local recurrence within 5 years of radiotherapy and 50 samples from patients who achieved long-term recurrence-free survival (**Figure 5A**). Our results indicated that high HOXA1 expression was significantly correlated with poor overall survival and high recurrence risk of NPC after radiotherapy (**Figure 5B, C**). This confirmed that HOXA1 enhanced radioresistance of cancer cells. Moreover, we knocked down HOXA1 in CNE1 and HNE1 NPC cells using a siRNA targeted against HOXA1. HOXA1 knockdown significantly reduced the expression levels of EGFR, CDK6, and CAV1 (**Figure 5D, F**). We also assessed the proliferation capacity of the cells and found that HOXA1 knockdown reduced the proliferation of CNE1 and HNE1 cells (**Figures 5E, G**). Next, we detected the radiosensitivity of these cells via colony formation assays. The dose–survival curves suggested that HOXA1 knockdown significantly sensitized these NPC cells to irradiation (**Figures 5H, I**).

### HOXA1 knockdown attenuated the DNA repair capacity of NPC cells

**Figure 6:**
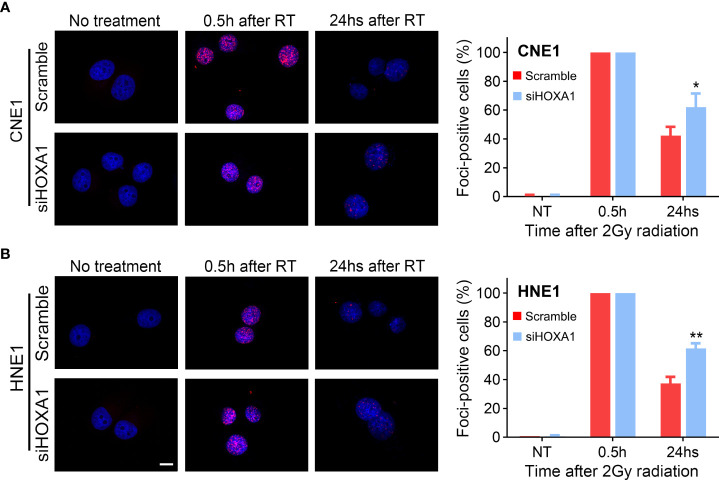
HOXA1 affected DNA repair capacity of NPC cells. enhanced the radioresistance of NPC cells. Immunofluorescence staining for γH2AX indicated that HOXA1 knockdown delayed absorption of γH2AX foci induced by 2Gy irradiation in CNE1 **(A)** and HNE1 cells **(B)**. **(B)** Cumulative bar chart showing that high HOXA1 expression was correlated with high risk of local relapse in NPC patients. Representative immunofluorescence images and quantification of the percentage of γH2AX foci-positive cells were shown. A positive cell was defined by the presence of more than 10 γH2AX foci. Scale bars, 10 μm. *P < 0.05, **P < 0.001.

DNA double-strand breaks (DSBs) are the main lesions induced by irradiation; therefore, capacity to repair DSBs is closely related to radiosensitivity (19). In addition, our results indicated that several genes associated with DNA repair were enriched in the samples with high HOXA1 expression (**Figure 4A**). Thus, we detected the DNA damage response induced by 2 Gy irradiation in control and HOXA1-knockdown NPC cells. We conducted immunofluorescence assays 0.5 and 24 h after irradiation to examine the phosphorylation of H2A.X at Ser139 (γH2AX, a biomarker of DSBs). The results showed that HOXA1 knockdown did not affect formation of γH2AX foci induced by irradiation. However, HOXA1 knockdown significantly delayed absorption of γH2AX foci (**Figure 6**). These results suggest that HOXA1 could affect radiosensitivity by regulating DNA repair.

## Discussion

In this study, we found that HOXA1 has potential to serve as a radioresistance-predictive biomarker in multiple cancer types. We found that high HOXA1 expression was associated with poor prognosis and high recurrence risk in HNSCC and LGG patients who had undergone postoperative radiotherapy, and in NPC patients who had undergone radical radiotherapy, whereas HOXA1 had no predictive value with respect to prognosis in patients who underwent surgery alone. Many studies have reported that elevated HOXA1 expression is correlated with poor prognosis in multiple cancer types (30, 31). However, little was known about whether high HOXA1 expression promotes the radioresistance of cancer cells. Our study provides novel insights that could inform further research on the role of HOXA1 in radioresistance.

HOXA1 is a well-reported oncogene that is upregulated in human malignancies including HNSCC, breast cancer, and non-small-cell lung cancer (17, 32, 33). Nevertheless, the mechanism underlying the upregulation of HOXA1 in cancer cells was not completely clear. There is no evidence that mutation leads to the abnormal HOXA1 expression observed in tumor tissues. However, epigenetic modifications of the HOXA1 promoter could be a mechanism that induces abnormal expression of HOXA1. In GBM, long noncoding RNA HOTAIRM1 was shown to mediate histone methylation modifications in the promoter region that increased HOXA1 expression (30). In HNSCC, studies have reported that HOXA1 expression levels were negatively correlated with the DNA methylation level of the HOXA1 promoter (17).

We also performed *in vitro* experiments to confirm the radiosensitivity regulation function of HOXA1 in NPC cells. Our results confirmed that knockdown of HOXA1 expression significantly sensitized NPC cells to irradiation. Few studies have reported that HOXA1 regulates the radiosensitivity of cancer cells. However, it has been reported that high HOXA1 expression is correlated with cisplatin resistance of lung adenocarcinoma (34). In addition, many studies have reported that HOXA1 promoted proliferation and invasion of different cancer types (13, 30, 35). In our study, we also observed that HOXA1 knockdown reduced the proliferation capacity of NPC cells, consistent with previous reports.

Mechanistically, HOXA1 could regulate radiosensitivity via effects on the DNA repair capacity of cancer cells. Our results indicated that several terms associated with DNA repair were significantly enriched in HOXA1 high expression samples. *In vitro* experiments also demonstrated that knockdown of HOXA1 expression hindered the repair of DNA DSBs induced by 2 Gy irradiation in NPC cells. Previous studies reported that expression of some metabolism-related genes was correlated with radiosensitivity in HNSCC (36, 37). In this study, we also found that the oxidative phosphorylation gene set was significantly negatively enriched in HOXA1 high-expression samples in both HNSCC and LGG. It had been reported that enhancing oxidative phosphorylation by reversing the Warburg effect sensitized glioma cells to irradiation (29). Thus, HOXA1 may also induce radioresistance via effects on oxidative phosphorylation.

In addition, we found that HOXA1 regulated the expression of some radioresistance-associated genes including EGFR, CDK6, and CAV1. Our results suggest that HOXA1 expression is positively correlated with expression of EGFR, CDK6, and CAV1 in both HNSCC and LGG. Knockdown of HOXA1 also decreased the expression of these three genes in NPC cells. Moreover, we found some putative HOXA1 binding sites in the promoter regions of these three genes using the JASPAR database (**Supplementary Table 2**). Studies have reported that these three genes were associated with radioresistance. EGFR induced radioresistance of glioma cells via triggering the PI3K–Akt and MEK–ERK pathways and attenuated cetuximab-mediated radiosensitization of squamous cell carcinoma cells via the JIP-4/JNK2 signaling pathway (26, 38). CDK6 expression was correlated with radioresistance in HNSCC and NPC. Inhibition of CDK6 or targeting CDK6-associated signaling pathways enhanced the radiosensitivity of HNSCC or NPC cells (27, 39). CAV1 increased oxidative stress protection and DNA repair, and its expression was correlated with radioresistance in rhabdomyosarcoma, pancreatic cancer, and lung cancer (28, 40, 41).

Taken as a whole, our data suggest that HOXA1 has potential as a predictive marker for intrinsic radioresistance in HNSCC, LGG, and NPC. Mechanistically, HOXA1 affected radiosensitivity via regulation of the DNA repair capacity of cancer cells. Knockdown of HOXA1 attenuated the DNA repair capacity and enhanced the radiosensitivity of NPC cells. Our results suggest that HOXA1 could be used to predict radioresistance and guide individualized treatment in multiple cancer types. However, more detailed basic and clinical studies are needed to confirm the regulatory function and clinical applications of HOXA1.

## Data availability statement

The original contributions presented in the study are included in the article/**Supplementary Material**. Further inquiries can be directed to the corresponding authors.

## Author contributions

YY and JL designed the study and experiments. LH wrote the manuscript. LH, ML and WG carried out the experiments. LH and ML performed the data analysis. All authors contributed to the article and approved the submitted version.

## Funding

This study was supported by Guangzhou Municipal Science and Technology Project (202201010965), National Natural Science Foundation of China (81602067), and Guangzhou Key Medical Discipline Construction Project.

## Conflict of interest

The authors declare that the research was conducted in the absence of any commercial or financial relationships that could be construed as a potential conflict of interest.

## Publisher’s note

All claims expressed in this article are solely those of the authors and do not necessarily represent those of their affiliated organizations, or those of the publisher, the editors and the reviewers. Any product that may be evaluated in this article, or claim that may be made by its manufacturer, is not guaranteed or endorsed by the publisher.
