# Supplementary material for: HOXA1 is a radioresistance marker in multiple cancer types
Source: Front Oncol. 2022 Sep 2;12:965427. doi: 10.3389/fonc.2022.965427 (PMC9478604; doi:10.3389/fonc.2022.965427)
Supplement: Supplementary file 1 [file Table_1.docx]

Supplementary table 1 Correlation of HOXA1 expression with clinical parameters of NPC patients.

| Characteristics | n | | HOXA1 | | *P*-value |
| --- | --- | --- | --- | --- | --- |
|  |  |  | Low | High |  |
| Age (y) | |  |  |  |  |
| ≤ 45 | 34 | | 18 (52.9%) | 16 (47.1%) | 0.147 |
| > 45 | 36 | | 12 (33.3%) | 24 (66.7%) |  |
| Sex |  | |  |  |  |
| male | 57 | | 26 (45.6%) | 31 (54.4%) | 0.371 |
| female | 13 | | 4 (30.8%) | 9 (69.2%) |  |
| T stage |  | |  |  |  |
| T1-2 | 42 | | 25 (59.5%) | 17 (40.5%) | **0.001** |
| T3-4 | 28 | | 5 (17.9%) | 23 (82.1%) |  |
| N stage |  | |  |  |  |
| N0 | 47 | | 17 (36.2%) | 30 (63.8%) | 0.128 |
| N1-3 | 23 | | 13 (56.5%) | 10 (43.5%) |  |
| Recurrence |  | |  |  |  |
| No | 50 | | 26 (52.0%) | 24 (48.0%) | **0.017** |
| Yes | 20 | | 4 (20.0%) | 16 (80.0%) |  |
