# Supplementary material for: HOXA1 is a radioresistance marker in multiple cancer types
Source: Front Oncol. 2022 Sep 2;12:965427. doi: 10.3389/fonc.2022.965427 (PMC9478604; doi:10.3389/fonc.2022.965427)
Supplement: Supplementary file 2 [file Table_2.docx]

Supplementary table 2 Putative HOXA1 binding sites in the promoter regions of EGFR, CDK6 and CAV1 using the JASPAR database.

| Gene | Score | | Relative score | Predicted sequence |
| --- | --- | --- | --- | --- |
| EGFR | | 6.308 | 0.903 | ataatggc |
| CDK6 | | 8.989 | 0.954 | ataattat |
| CDK6 | | 6.773 | 0.912 | ataattgt |
| CAV1 | | 7.836 | 0.932 | tcaattag |
| CAV1 | | 6.773 | 0.912 | acaattat |
