# Supplementary figures and images for: HOXA1 is a radioresistance marker in multiple cancer types
Source: Front Oncol. 2022 Sep 2;12:965427. doi: 10.3389/fonc.2022.965427 (PMC9478604; doi:10.3389/fonc.2022.965427)

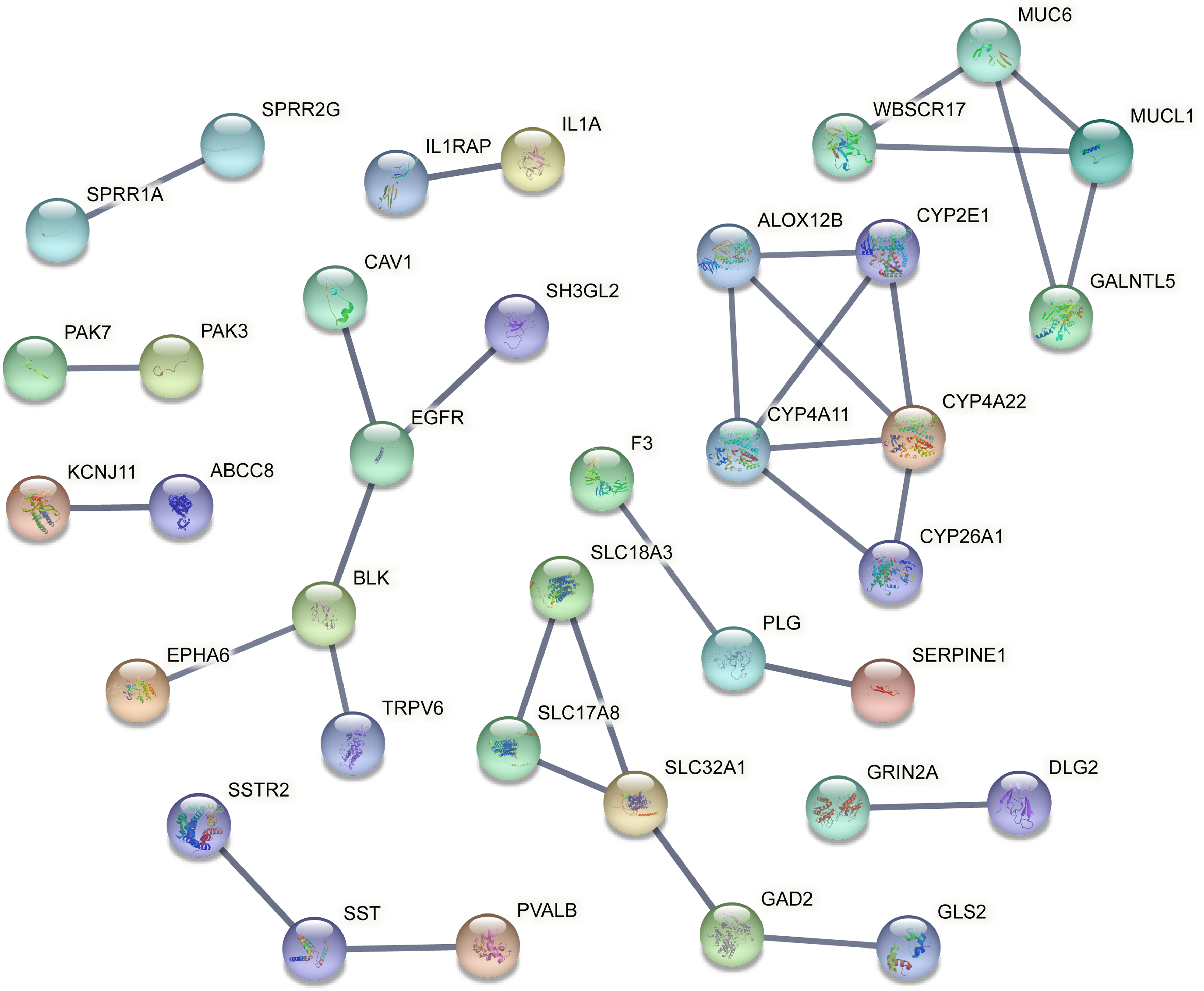

Supplement: Supplementary file 3 [file Image_1.tif]
